# Amplification-Free Strategy for miRNA Quantification in Human Serum Using Single Particle ICP–MS and Gold Nanoparticles as Labels

**DOI:** 10.1021/acs.analchem.4c01904

**Published:** 2024-07-19

**Authors:** Sara González Morales, Carlos López-Portugués, Manuel Fernández-Sanjurjo, Eduardo Iglesias-Gutiérrez, María Montes Bayón, Mario Corte-Rodríguez

**Affiliations:** † Department of Physical and Analytical Chemistry, Faculty of Chemistry, University of Oviedo, Julián Clavería 8, 33006 Oviedo, Spain; ‡ Department of Functional Biology (Physiology), University of Oviedo, Julián Clavería s/n, 33006 Oviedo, Spain; § Health Research Institute of the Principality of Asturias (ISPA), Av. Hospital Universitario s/n, 33011 Oviedo, Spain

## Abstract

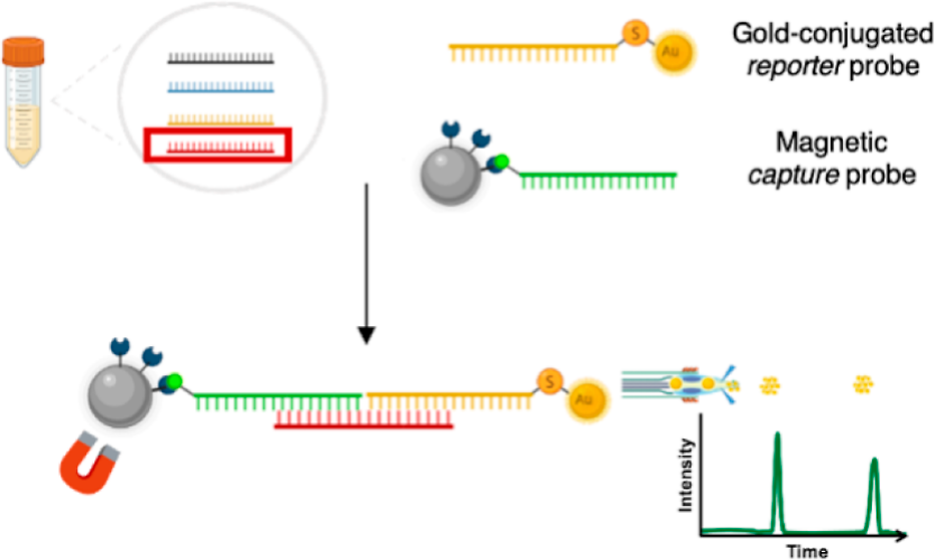

MicroRNAs (miRNAs), which are short single-stranded RNA sequences between 18 and 24 nucleotides, are known to play a crucial role in gene expression. Changes in their expression are not only involved in many diseases but also as a response to physiological changes, such as physical exercise. In this work, a new analytical strategy for the sensitive and specific analysis of miRNA sequences in human plasma is presented. The developed strategy does not depend on any nucleic acid amplification process and can be obtained in direct correlation to the number of events obtained by using single-particle ICP–MS measurements. The high selectivity of the assay (up to single nucleotide polymorphisms) can be achieved by a double hybridization process of the target miRNA with a complementary capture oligonucleotide that is conjugated to a magnetic microparticle and simultaneously with a complementary reporter oligonucleotide conjugated to a gold nanoparticle. Thanks to the novel approach followed in this method, the stoichiometry of the oligonucleotide-nanoparticle conjugates does not need to be addressed for the quantification of the target miRNA, which also represents a big advantage over other similar methods. The optimized method is applied to the determination of a miRNA as a biomarker of physical exercise in non-spiked human serum samples, and the results are validated against rt-qPCR. The achieved sensitivity permits the direct differentiation among sedentary and sportive subjects. This general platform can be easily applied to any other sequence by only modifying the capture and reporter oligonucleotides, paving the way for multiple clinically interesting applications.

## Introduction

Microribonucleic acids (miRNAs) are small noncoding RNA biomolecules with a length between 18 and 24 nucleotides that are involved in different cellular processes, such as gene expression modulation. In fact, the main function of miRNAs consists of their union with mRNAs by base complementarity, either preventing their translation or inducing their degradation, resulting in both cases in a negative post-transcriptional regulation of gene expression. Over 60% of protein-coding genes are known to be regulated by different miRNAs.^1^

Although miRNAs mainly play their role in intracellular compartments, circulating extracellular miRNAs (c-miRNAs) have been also stably detected in different body fluids, including blood and urine.^2,3^ This has led over the past few years to study the role of c-miRNAs as communicators between tissues and as minimally invasive biomarkers in numerous physiological and pathological situations, ranging from the response to exercise^4^ or the changes during pregnancy^5^ to tumoral^6,7^ or cardiovascular diseases.^8,9^ Therefore, the specific and sensitive determination of miRNAs is highly demanded due to their multifactorial practical implications.

In this regard, the quantification of miRNAs is especially challenging due mainly to the wide concentration ranges in which they may be present and their small size. In general, miRNA determination can be done either by including a previous step of amplification [for example using reverse transcription polymerase chain reaction (RT-PCR),^10^ hybridization methods (microarrays),^11^ or sequencing strategies^12,13^]. The use of RT-PCR is considered to be the gold standard for miRNA determination. However, the intrinsic nature of miRNAs regarding their very small size and the high homology between different sequences of miRNAs from the same family^14^ makes RT-PCR prone to generating false-positive results. Additionally, many PCR-based approaches do not provide absolute concentrations but relative amounts to establish whether a sample has a higher or lower expression of certain miRNAs compared with a previous condition.

On the other hand, some miRNA analysis methods rely on nucleic acid hybridization^14^ of complementary nucleotides between the target miRNA sequence and another RNA or DNA sequence, forming a double-stranded molecule. These hybridization methods include microarrays in solid phase, but also liquid phase approaches that are more useful for in vivo studies. Hybridization is often recognized by electrochemical or spectroscopic methods,^14^ which usually need to make use of electroactive (e.g., metallic nanoparticles) or luminescent tags, respectively. As in the case of RT-PCR, miRNA in situ hybridization has been challenging because of the small sizes of miRNAs (∼22 nucleotides) and, thus, low selectivity.

Metal nanoparticles can be used as tags that allow to amplify the response of the detection methods, sometimes helping to avoid time-consuming and prone-to-error amplification strategies.^15^ In this regard, electrochemical methods are especially benefited by the use of these metallic nanostructures, but they have also been applied for spectroscopic detection of miRNA based on their outstanding optical properties. Some examples include colorimetric methods,^16−19^ light scattering,^20^ or changes in the surface-plasmon resonance properties of gold nanoparticles.^21^ All of these methods rely on measuring changes of the optical properties of nanoparticles in the presence of the analyte. Metallic nanoparticles can also be advantageously used as tags when applying mass spectrometry (MS), particularly elemental MS, as a detection method.

While molecular metal labeling strategies can add up to a few tens of heteroatoms to the detection probes, nanoparticles can be used as labels that add several thousands of atoms per labeled molecule, and this can become especially useful when using elemental detectors like ICP–MS, which is mass dependent. Due to this advantageous principle, some studies have made use of quantum dots and silver nanoparticles,^22^ gold nanoparticles,^23^ or NaLnF_4_ nanostructures^24^ to determine miRNAs. Other authors have used lanthanide tags for the detection of DNA or RNA sequences.^25−27^ All of these mentioned strategies rely on the final measurement of the metal ions released in the solution after digestion, which is proportional to the concentration of target miRNA. Some other strategies release the metal after performing enzyme-free amplification by means of entropy-driven catalytic amplification (EDC). In the presence of target miRNAs, EDC amplification occurs, releasing lanthanide-labeled reporter strands that are then quantified by ICP–MS.^28^

A few studies apply the novel operating mode of ICP–MS that is based on the measurement of single nanoparticles, which is known as single-particle-ICP-MS (SP-ICP-MS) for miRNA analysis. Using metal nanoparticles as labels, they can be detected individually or as aggregates by this technique, which leads to two main approaches. One of them aims to study the formation of aggregates caused by the presence of the analyte sequence in a sample. This approach has been used to detect DNA sequences,^29^ SARS-CoV-2, influenza,^30^ and miRNAs by triggering the activity of a multicomponent nucleic acid enzyme (MNAzyme).^31^

The second approach implies using SP-ICP-MS to “count” the number of nanoparticles used as labels for specific nucleic acid sequences. One of these applications was achieved for three different DNA sequences in a heterogeneous assay by hybridization with Au, Pt, or Ag nanoparticles.^32^ Indirectly, Jiang et al.^33^ applied this idea to count the number of gold nanoparticles released after a hybridization sandwich assay to detect the activity of an uracil-DNA glycosylase. Similarly, Xu et al.^34^ used a hybridization sandwich assay and gold nanoparticles to quantify O157:H7 16S rRNA from *Escherichia coli* in food. This work uses magnetic particles to capture the analyte RNA sequence and gold nanoparticles as reporting labels, which are counted by SP-ICP-MS.

Although these strategies have been applied to the detection and quantification of DNA and RNA sequences, very few have been used for miRNA quantification. miRNA is even more challenging as an analyte because of its easy degradation, short sequence length (typically 22 nucleotides), and challenging extraction procedures. One strategy has been proposed that combines the aggregation of gold nanoparticles with the particle-counting approach.^35^

**Scheme 1:**
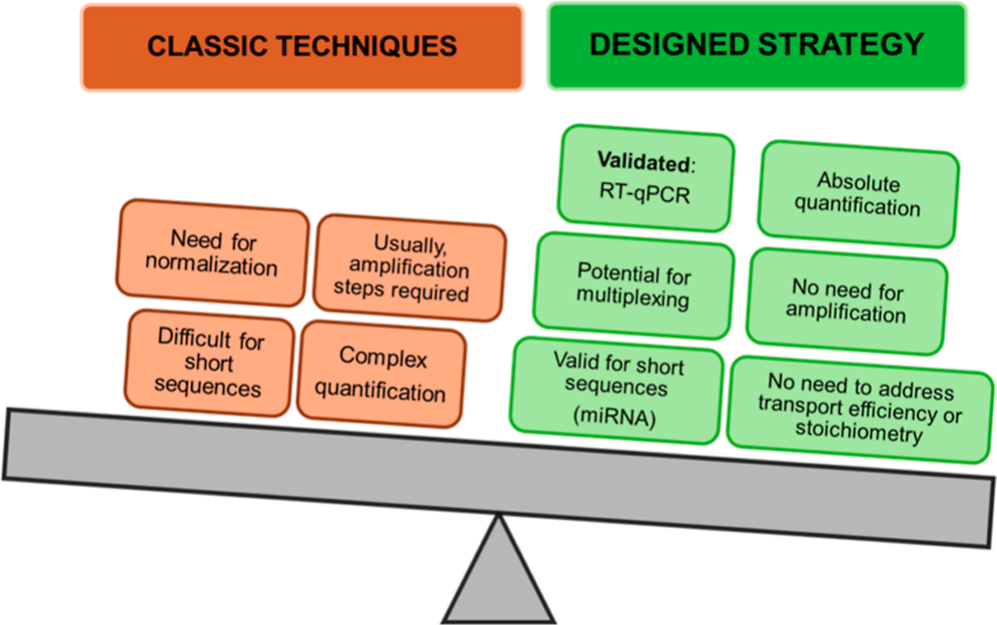
Main Differences of the Methodology Proposed in This Article in Comparison With Other Traditional Techniques

In this work, miRNA quantification is based on the specificity of nucleic acid hybridization and the highly sensitive and specific nanoparticle counting capabilities of individual nanoparticles by SP-ICP-MS. The amplification-free workflow that we propose is based on the hybridization of a specific target miRNA sequence (miR-16-5p) with two DNA sequences that are each complementary to half of the target, respectively. One of them is conjugated to a magnetic microparticle (capture probe) that is used for the enrichment and washing of the target sequence. The other half-complementary sequence is conjugated to a gold nanoparticle that is detected by SP-ICP-MS (detection probe). Although the analytical strategy is comparable to this from Xu et al.,^34^ it is important to note that our methodology is applied to quantify different levels of expression of miRNA sequences as short as 22 nucleotides that are naturally present in human serum samples. Scheme 1 summarizes the main differences between the proposed strategy and other methods for miRNA quantification. Therefore, this methodology represents a step forward in facing challenges like miRNA pre-concentration in a complex matrix with the important role of quantification, which is crucial to extracting conclusions depending on the expression levels.

## Experimental Section

### Instrumentation

The measurements were performed by using the iCAP TQ triple quadrupole ICP–MS system from Thermo Fisher Scientific. All measurements, including single particle ICP–MS measurements, were done using the standard configuration for sample introduction, including the MicroMist nebulizer at a sample flow rate of 0.4 mL min^–1^ and a cyclonic spray chamber because a high transport efficiency was not crucial for this application. The sample introduction system was operated in combination with an ASX-560 autosampler from Teledyne Cetac. The ICP–MS was tuned daily to maximize sensitivity while keeping the formation of oxides and doubly charged species below 3 and 5%, respectively.

For spectrophotometric measurements of nucleic acid concentrations, a NanoDrop spectrophotometer (Thermo Fisher Scientific) was used. A transmission electron microscope MET JEOL-JEM-2100F was used to obtain images of the nanoparticles. A scanning electron microscope MEB JEOL-6610LV with EDX microanalysis was used for the images and elemental analysis of microparticles. Other basic laboratory instrumentation was also used, including an analytical precision balance, an ultrasonic bath, a vortex mixer, and a block heater.

### Materials and Reagents

All solutions were prepared in ultrapure water using a PURELAB flex 3 apparatus from Elga Veolia. For solutions needed to preserve miRNA samples, DEPC-treated water was purchased from Ambion and Life Technologies. Low protein binding microcentrifuge tubes from ThermoFisher Scientific were used to minimize the level of RNA-unspecific binding to plastic surfaces. To prevent RNase contamination, RNaseZap from Thermo Fisher Scientific was used as a cleaning agent.

DNA oligonucleotides were custom-made by Invitrogen. They were shipped as a lyophilized powder and reconstituted, as indicated by the manufacturer. The gold nanoparticles used for conjugation were 22 nm citrate carboxy pegylated nanoparticles OD10 and GC-PEG-20-1 from Nanovex Biotechnologies. Magnetic microparticles were kindly provided by Dr. Elena Añón from the University Hospital of Asturias as part of the ferritin determination kit Elecsys Ferritin II Gen, from Roche.

Tris buffer saline (TBS) was purchased as a soluble tablet from Sigma-Aldrich. Phosphate buffer and aging buffer were prepared in-house using inorganic salts from Merck. Tween 20 was obtained from Sigma-Aldrich, as was the tris(2-carboxyethyl)phosphine (TCEP) 0.5 mol L-1 for the deprotection of the thiolated oligo.

The gold nanoparticles of quality control material LGCQC5050 from LGC Standards were used to determine transport efficiency. Ionic gold 1000 ppm standard NIST3121 from Merck was used for calibration. All ionic solutions were diluted in 2% nitric acid prepared from 65% HNO_3_ from Acros Organics, previously purified by sub-boiling distillation.

Argon gas for the operation of ICP–MS with 99.999% purity was supplied by Air Liquide.

### Single Particle ICP–MS Measurement

Single particle ICP–MS analysis (SP-ICP-MS) was used as a tool for the characterization of gold nanoparticles and for counting the gold nanoparticles during the assay. For nanoparticle characterization, the intensity of the gold events was transformed into the mass of gold per nanoparticle via calibration with inorganic gold standards, as previously reported. The transport efficiency was measured daily following the particle number method^36^ using the standard 30 nm gold nanoparticles material LGCQC5050 from LGC Standards. This colloidal AuNPs, with a stated initial concentration of 1.47 × 10^11^ particles mL^-1^, was appropriately diluted to 3.0 × 10^4^ particles mL^–1^ in water. Typical values for transport efficiency were around 5%. It must be noted that no special sample introduction system with high transport efficiency was used. The sample flow rate was gravimetrically determined every measurement day, resulting in typical values close to 0.2 mL min^–1^. Unless otherwise specified, measurement time was always 2 min. Therefore, all references to the number of events in this work correspond to 2 min of signal acquisition.

As previously described, data treatment for AuNP characterization was performed using an in-house prepared Microsoft Excel spreadsheet that extracts from the whole data set those events that are higher than the background plus 3 times its standard deviation by means of an iterative process. A similar strategy was used to count the number of nanoparticles after the assay, which is related to the concentration of the analyte miRNA in the sample. In this case, the threshold was manually set in order to simplify the calculations. When the events are counted, their intensity is not important anymore. Therefore, manual thresholding is a valid strategy when counting events with an almost inexistent background, as can be seen in Figure S1.

For the characterization of the gold nanoparticles used as labels, the event intensity was correlated with the mass of gold per nanoparticle through a calibration curve constructed with gold elemental standards and taking into account their transport efficiency, the sample flow rate, and the dwell time, as previously described.^37^

### Oligonucleotides

Three DNA oligonucleotide sequences were used throughout this study: the target analyte miR-16-5p (1), the biotinylated half-complementary sequence to the 3′ end (capture probe) (2), and the thiolated half-complementary sequence to the 5′ end (detection probe) (3). The target analyte, miR-16-5p (1), was chosen because it is highly expressed in human plasma/serum.^38^ In addition, several authors have described that its plasma concentration is modified in response to acute physical exercise and training.^39^

All optimization steps were performed to avoid degradation problems associated with the manipulation of RNA using the equivalent DNA sequence for miR-16-5p (1*), since hybridization of RNA with DNA is also efficient. For this reason, reporter and capture probes were kept as DNA even when using real miRNA samples.

The complementary sequences were elongated with seven AAA triplets (total 21 A) for the capture probe and eight triplets (24 A) for the reporter sequence. This elongation serves to separate the complementary region from the labeling group, avoiding any steric impairments. All sequences are specified in Table S1.

### Functionalization of Gold Nanoparticles

4.5 nmol of the reporter oligo dissolved in phosphate buffer were incubated with 3 μL of a solution containing 0.5 mol L^–1^ tris(2-carboxyethyl)phosphine (TCEP) in water for 2 h at room temperature. This step allows the reduction of the protected thiol group of the oligonucleotide. During this time, 25 μL of PEG stabilized gold nanoparticles (6.7 × 10^12^particles mL^–1^) were precipitated by centrifugation at 10,000 rpm for 5 min, removing the supernatant and subsequently resuspended in a buffer at pH 6.0 containing 10 mmol L^–1^ citrate and 0.01% Tween-20. This suspension was mixed with 10 μL of the previously reduced oligo and incubated for 30 min at room temperature.

With the aim of increasing the binding efficiency and avoiding the aggregation of the nanoparticles, the ion strength of the solution was gradually increased by serial additions of 2 mol L^–1^ NaCl and 0.01% Tween-20, until a final concentration of NaCl was 0.3 mol L^–1^, which was achieved after 4 additions. Each buffer addition was followed by the application of 1 min in an ultrasonic bath and 20 min gentle shaking at room temperature. This process is usually referred to as aging in the literature.

After the aging, the oligo-conjugated gold nanoparticles (detection probe) were washed by centrifugation for 5 min at 10,000 rpm and resuspension in 500 μL of TBS. The number of washing steps was optimized to four.

### Preparation of the Capture Probe

The capture probe was prepared by conjugating the capture biotinylated oligo with streptavidin-coated magnetic microparticles. Following the instructions of the manufacturer, 65 μL of magnetic beads were washed three times using a magnet and a washing buffer with 2 M NaCl, 1 mM EDTA, and 10 mM Tris in ultrapure water at a pH of 7.5. 92 pmol of oligo were incubated with the microparticles for 20 min at room temperature. The excess oligo was then washed away with 2 washing steps using an external magnet to retain the conjugated magnetic microparticles.

### Sample Pre-Treatment

Serum samples from anonymous professional athletes and sedentary subjects were obtained by centrifugation of coagulated blood for 15 min at a rate of 2500 rpm. Blood samples were collected using standardized techniques and materials from an antecubital vein under fasted conditions at least 12 h after the last exercise bout. Sample pretreatment, as well as all steps of the assay, were performed taking all precautions to work in RNase-free conditions, including the use of DEPC-treated ultrapure water, a double pair of gloves, sterile filter pipet tips, etc. These serum samples were deep-frozen and kept at −80 °C until the assay. Samples were obtained from three volunteers with no known pathology, with special emphasis on cardiovascular disease, and in agreement with the Research Ethics Committee of the University of Oviedo (reference 124/17). Two volunteers were self-considered with a low physical activity, which was then confirmed by using the Global Physical Activity Questionnaire (GPAQ). Sedentary volunteers were one man and one woman with ages between the third sample was randomly selected from a sample collection of eight elite male athletes (26.2 ± 3.6 years) from the Spanish National Kayaking Team, which had been previously obtained and preserved in another study.^40^

### SP-ICP-MS for Characterization of Naked Nanoparticles

The characterization of the gold nanoparticles was carried out by SP-ICP-MS. Briefly, the dwell time of the ICP–MS was reduced to 5 ms, and the samples were diluted enough to decrease the probability of detecting more than one particle at the same time. The signal intensity of the transient events caused by the single nanoparticles arriving at the ICP–MS was then transformed into the mass of gold by means of an external calibration curve of ionic gold and taking into account the transport efficiency of the ionic standards, which was calculated daily using the LGCQC5050 nanomaterial. Once the mass of gold per nanoparticle was obtained, knowing the spherical geometry of the particles and their composition of pure gold, the volume and, therefore, the diameters were calculated.

### miRNA Determination Global Assay

In a typical optimized assay, the capture and detection probes were freshly prepared the day before. 100 μL of the sample is diluted to 500 μL in TBS. In the case of calibrations, the sample is a blood serum pool, which is spiked with the corresponding volume of surrogate DNA target sequence. Then, 26 μL of detection probe and 144 μL of capture probe are added and incubated at 70 °C for 10 min to denature any hybridization or secondary structures of the probes or the analyte. This temperature is higher than the melting point of all the used oligos but lower than 80 °C, which could cause denaturation of biotin. The mixture is then allowed to cool slowly to room temperature for 3 h to guarantee specific hybridization, washed twice with 500 μL of TBS, twice with 300 μL of TBS, and finally resuspended in 300 μL of TBS.

Before the measurement, the formed sandwich is heated up to 97 °C for 10 min, causing the denaturation of all nucleic acid hybrids as well as the biotin–streptavidin interactions. This separates the gold nanoparticles from the magnetic beads, which can be magnetically removed. The resulting gold nanoparticle suspension is then adequately diluted and measured by SP-ICP-MS.

### Evaluation of Physical Activity

The level of physical activity of healthy volunteers was determined using the GPAQ developed by the World Health Organization (WHO) to evaluate physical activity in all countries, collecting information about the physical activity in three domains divided into 16 questions: activity at work, travel to and from places, and recreational activities. The combination of all the information in the questionnaire as explained in the evaluation guidelines^41^ provides the value of the continuous indicator known as MET-minutes per week, or time spent in physical activity, as an equivalent combination of moderate- and vigorous-intensity physical activity. WHO recommendations on weekly physical activity are at least 600 MET-minutes, which equals 150 min of moderate physical activity or 75 min of vigorous physical activity. In this work, both sedentary volunteers were below 700 MET-minutes. The professional athlete was not evaluated by the GPAQ.

### miRNA Quantification by RT-qPCR

The concentration of target miRNA was quantified by RT-qPCR as the gold standard for miRNA quantification to validate the proposed methodology. Total circulating RNA from 200 μL of serum was isolated using the miRNeasy Serum/Plasma Advanced Kit (Qiagen) following the manufacturer’s instructions. RNA was eluted in 20 μL RNase-free H_2_O. For miRNA retrotranscription, cDNA was synthesized using the miRCURY LNA RT Kit (Qiagen). Briefly, 2 μL of RNA was reverse transcribed in 10 μL of reaction with the following conditions: incubation for 60 min at 42 °C, heat-inactivation for 5 min at 95 °C, and immediately cooling to 4 °C. For quantification, cDNA was diluted 20× and 4 μL was used in 10 μL qPCR reactions with a miRCURY LNA SYBR Green PCR Kit (Qiagen) on a 7900HT fast Real-Time PCR System (Applied Biosystems) with the following cycling conditions: 2 min at 95 °C, 40 cycles of 10 s at 95 °C and 1 min at 60 °C, followed by a melting curve analysis.

In the quantification, a standard curve was created by triplicate serial dilutions of UniSp2 (2, 0.2, 0.02, and 0.002 pmol mL^–1^). Then, hsa-miR-16-5p was analyzed in triplicate in the same samples previously described. SDS v2.3 software was used for both the determination of the quantification cycle (Ct) and the melting curve analysis. Ct was defined as the fractional cycle number at which the fluorescence exceeded a given threshold. The specificity of the PCR reaction was corroborated by a melting curve analysis. Has-miR-16-5p quantification was performed using the standard curve created for this purpose.

## Results and Discussion

### Characterization of Naked and Conjugated Gold Nanoparticles

**Figure 1:**
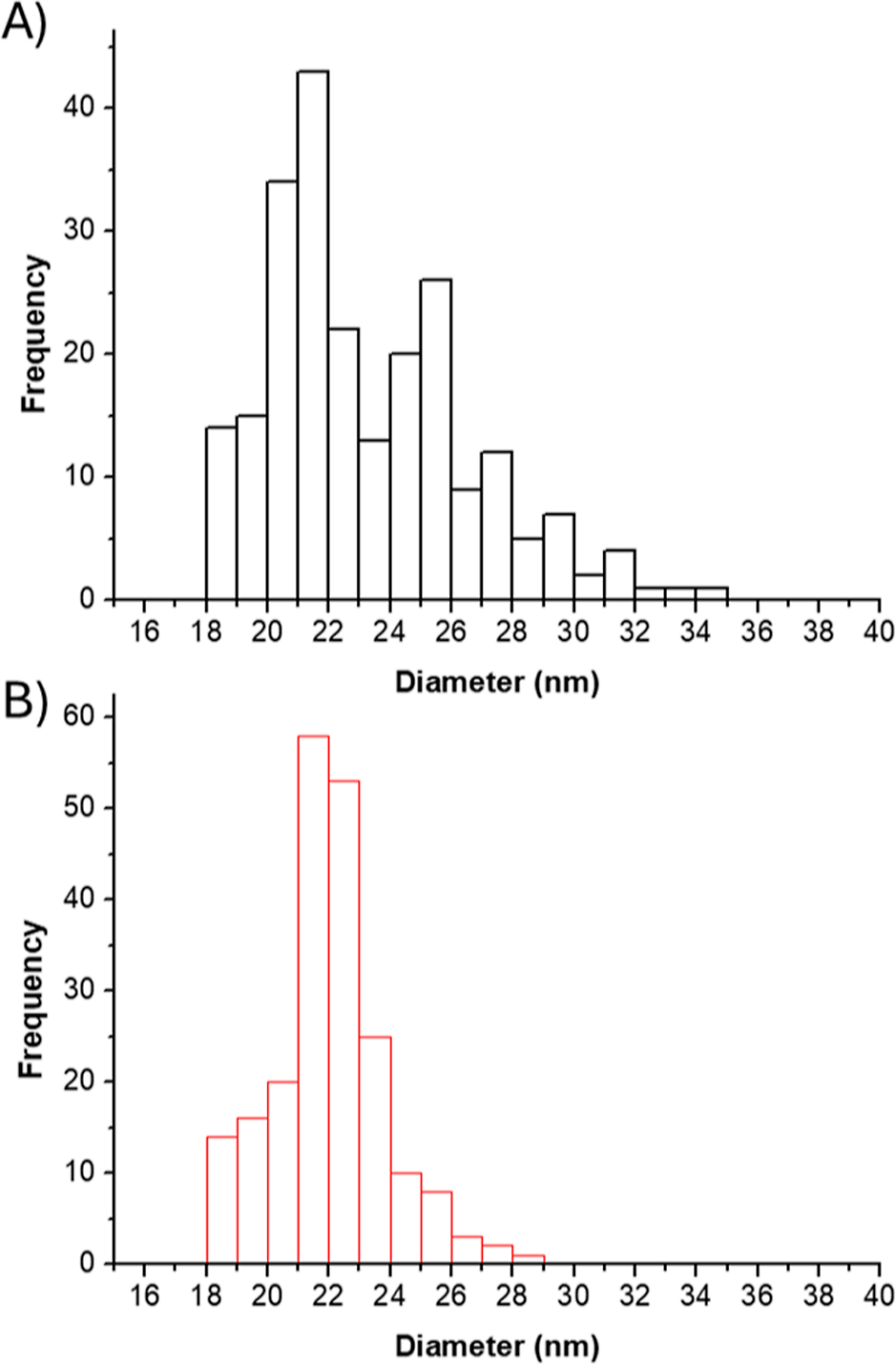
Histograms showing the diameters distribution of the gold nanoparticles analyzed by SP-ICP-MS. (A) (in black) Unconjugated nanoparticles and (B) (in red) nanoparticles after being conjugated with the oligonucleotide probe, with a shift to higher diameter values.

The gold nanoparticles used as metal labels for the detection probe were characterized both before and after their conjugation with the DNA oligo by SP-ICP-MS, transmission electron microscopy (TEM), and DLS. The results of SP-ICP-MS, shown in Table 1 and Figure 1, reveal that the obtained mean diameters were 23.3 ± 3.3 nm, which was slightly larger than the value provided by the manufacturer (22 nm). When a Student-*t* test was performed to compare the mean diameters of the conjugated and original nanoparticles, no statistical differences between them or compared to the value given by the manufacturer was observed at a 95% confidence level. Under these conditions, the size limit of detection was calculated as 18 nm, well below the size of the nanoparticles. Both sets of particles showed a narrow distribution, as can be seen in Figure 1, although the nanoparticles showed a more monodisperse distribution after the conjugation (Figure 1B), probably due to an increased stability in solution when coated with negatively charged oligonucleotides.

**Table 1:** Theoretical (Specified by Manufacturer) and Experimental Diameter for the Pegylated Gold Nanoparticles Before (Bare) and After (Conjugated) Conjugation With the Oligo^a^

nanoparticles	manufacturer (nm)	experimental (nm)		
		SP-ICP-MS	DLS	TEM
original	20	23.3 ± 3.3	23.4	22.5 ± 2.1
conjugated	20	22.0 ± 1.8	46.0	23.0 ± 1.8

a Data shown as mean ± standard deviation of the dataset for the whole nanoparticle population.

**Figure 2:**
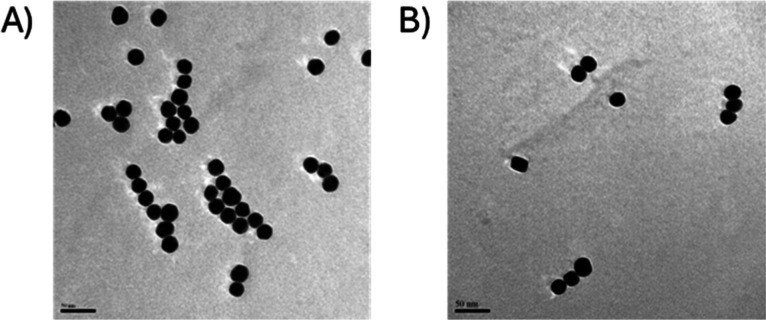
Representative TEM images of the gold nanoparticles used as labels. (A) Before and (B) after conjugation with the reporter oligonucleotide probe. The scale bar in all micrographs is 50 nm.

TEM was also used to observe and measure these nanoparticles. Figure 2 shows representative TEM images of the nanoparticles before (2A) and after (2B) conjugation. The gold nanoparticles used as metal labels show spherical morphology and relatively low dispersion with regard to their diameter. Additionally, no significant differences could be detected between the diameters obtained by SP-ICP-MS and TEM using the Student-*t* test at a 95% confidence level. At least 100 particles were graphically measured in TEM images by using the image processing open-access software ImageJ. The results of these measurements are shown as histograms in Figure S2. Mean diameters and standard deviations are also shown in Table 1. A good correlation between the particle sizes obtained by SP-ICP-MS, DLS, and TEM can be observed with an average diameter of 23 nm along all techniques.

The increase in the hydrodynamic diameter observed by DLS is probably ascribed to the surface charge of the particles affecting their hydrodynamic volume after the conjugation. Additionally, DLS provided a polydispersity index (PDI) that measures the heterogeneity of the particle sizes as 0 for a perfectly uniform sample. In these particles, the PDI was 0.096 before the conjugation but increased to 0.249 after the conjugation with the oligo. This might indicate either a slightly higher aggregation of the nanoparticles or the existence of nanoparticles with different numbers of oligo molecules coating their surface. The combination of both effects could be discarded to contribute to the higher PDI in the conjugated particles.

### Optimization of the Conjugation and Characterization of the Assay

For optimization and proof of concept, miR-16-5p was selected as the model molecule in this work. This miRNA been has shown to respond to physical exercise, being overexpressed in sedentary people and repressed with regular physical activity,^42^ but not after acute exercise.^43^ In order to determine the best conditions for the conjugation of the gold nanoparticles with the detection probe, mixtures with increasing concentrations of the probe and maintaining constant the number of nanoparticles were studied. For these experiments, the conjugated nanoparticles were removed from the suspension by centrifugation, and the excess probe was quantified in the supernatant by UV spectrophotometry. The presence of an excess of probe in the supernatant after conjugation at a concentration below the initial concentration used for conjugation would demonstrate the success of the conjugation as well as the saturation of the nanoparticles with the probe. The results for these experiments can be seen in Table S2. In light of these results, the concentration of 6.62 ng μL^–1^ was chosen to ensure saturation of the bioconjugation while avoiding an unnecessary excess of oligonucleotides. The conjugation of the capture probe with the magnetic microparticle was optimized in a similar way (Table S3). In this case, a concentration of 517 ng μL^–1^ was chosen for this incubation since it is the first concentration level where there is an excess of oligo.

Controlling and determining the stoichiometry between the DNA detection oligo and the nanoparticle is often a difficult problem. In the case of the proposed strategy, the use of stoichiometry is not required for the quantification; however, it was studied in order to have a better understanding of the assay. For this aim, three independent conjugations were prepared and injected separately, in triplicate in the FIA-ICP-MS system, as explained in Text S1 and shown in Figure S3. The results provided a stoichiometry of 1400 ± 10 oligonucleotides per nanoparticle, which is a plausible stoichiometry if we assume a total of 13,720 possible binding sites on the surface of each nanoparticle (see Supporting Information Text S2).

Finally, since little data were available from the magnetic microparticles, scanning electron microscopy (SEM) was used to characterize them. These were uniform spherical nanoparticles of 2 μm diameter formed by a solid iron core covered by C and Si (Figures S4 and S5).

### SP-ICP-MS for the Detection of miRNA

SP-ICP-MS was used for the detection of miRNA hybrids, which were considered the triple sandwich formed between the target miRNA, its half-complementary capture oligo probe bound to the magnetic microparticle, and the half-complementary detection oligo probe bound to the gold nanoparticle. The number of gold nanoparticles detected after the whole hybridization, capture, and washing procedure should be proportional to the miRNA concentration in the solution.

**Figure 3:**
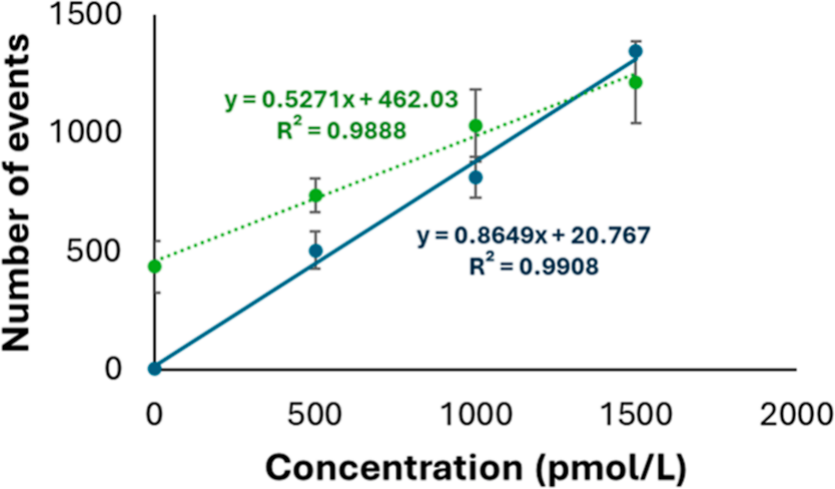
Calibration graphs used for the calculation of the relationship between the number of detected gold events using water (orange dotted line) and blood serum (blue solid line) as a matrix. Equations and correlation coefficients are shown next to and in the same color as the corresponding regression line. Error bars show the standard deviation of three replicates for each concentration level.

Initially, known concentrations of the target miRNA were spiked in water. After performing the assay, the number of gold (^197^Au^+^) spikes detected during a measurement time of 2 min was plotted against the target miRNA concentration as a calibration curve. The result of this calibration is shown in Figure 3 (green dotted line), with an acceptable linearity (*R*^2^ = 0.9888) and an equation for the correlation *N* = (0.53 ± 0.04)·*c* + (462 ± 37), with *N* being the number of events detected in a 2 min measurement and c the concentration of the analyte miRNA. After a 1000-fold dilution of the resulting solution from the assay, 400 events were still found in the blank. Such a result points toward a strong, unspecific adsorption of the gold nanoparticles that could not be avoided, even after careful cleaning in every step of the assay and optimization of reagent concentrations, incubation times, washing procedures, temperatures, and buffers (data not shown).

Several changes were tested along the protocol to decrease the unspecific adsorption of the nanoparticles that could be causing an increase in the background signals. The best signal/blank ratio was achieved when washing 4 times the formed sandwich in a compromise between reducing the background and maintaining good sensitivity (Figure S6).

When moving from a water matrix to miRNA-depleted blood serum, the linearity and reproducibility of the assay increased significantly, with a number of signals in the blanks below 10 events. This can be explained by the passivation of the surface of the gold nanoparticles upon the formation of a protein corona^44^ that prevents them from adsorbing onto the surface of the tubes and interacting with the magnetic beads and other species in the assay. The calibration curve obtained in the blood serum matrix is shown in Figure 3 (blue solid line) [*N* = (0.86 ± 0.06)·*c* + (20 ± 55)], in comparison to the graph obtained when the calibration was performed in water (green dotted line). There are statistical differences (*P* < 0.05) both in the slope and in the intersection of both calibration curves.

### Analytical Figures of Merit of the Assay

The limit of detection of this strategy was estimated as the concentration of target miRNA that is obtained by the detection of a minimum number of nanoparticles in the suspension that can be considered the particle number limit of detection. According to Laborda et al.,^45^ the minimum number of particles should be 15–30. Taking the highest limit of 30 nanoparticles, this corresponds to a limit of detection of 10.7 pmol L^–1^ as the miRNA concentration. This limit of detection in the low picomolar range is sufficient for some biological applications with high-expression miRNAs but could be easily decreased, for example, using higher sample amounts. A comparison with other DNA/RNA/miRNA quantification methods using SP-ICP-MS is shown in Table S4. Although our limit of detection is within the same order or higher than other published similar methods, it must be taken into account that (i) our strategy does not need any amplification or extraction step, (ii) our strategy was developed for miRNA quantification, although it is compared with DNA and long RNA sequence quantification strategies in Table S4, and (iii) we quantify miR-16-5p in a natural matrix where this miRNA should be present at different concentration levels. On the other hand, the size limit of detection of the nanoparticles did not change significantly from the previous experiments, and neither did the ionic gold background, keeping 18 nm as the size limit of detection.

The reproducibility of the assay, in terms of the relative standard deviation of a triplicate of the complete assay for every concentration of the analyte, ranges 7–12% (see every data point of Figure 3). It is important to establish that every data point of the calibration curve is obtained by using the same set of gold nanoparticles (with a given stoichiometry Au-probe) and magnetic microparticles (with a given stoichiometry microparticles-probe) and changing the analyte concentration (*X*-axis). Thus, every point in the calibration curve is affected by the stoichiometries (e.g., Au-probe and microparticle-probe) in the same extension, and this effect is shown in the calculated slope. Similarly, gold nanoparticle transport efficiencies to the ICP–MS do not need to be considered because they affect every data point of the calibration curve as well and will be included in the calculated slope.

Therefore, the slope of the calibration curve (number of events/miRNA concentration) accounts already for (i) stoichiometry of the AuNP-oligo conjugates and (ii) transport efficiency. This slope will be, of course, affected by these two factors. In fact, a higher transport efficiency will increase the slope with more events detected for the same concentration level, thus increasing the sensitivity of the assay. On the other hand, a higher number of probes per gold nanoparticle would decrease the slope (as more analyte molecules can hybridize on the same Au nanoparticle), which would be detected as only one event in the ICP–MS. Thus, as long as the calibration curve and the analysis of the samples are conducted within the same instrument configuration (same transport efficiency) and obtained with the same set of capture probes, no stoichiometry needs to be calculated.

### Selectivity of the Assay

In order to ensure that the developed assay would respond only to the target analyte, selectivity experiments were performed by using five different oligonucleotides with different degrees of similarity with the target. This is critical, taking into account single nucleotide polymorphisms affecting also miRNA sequences, and any of them lead to dysregulation of gene expression.^46−48^ The most different one (A) was chosen with a random nucleotide sequence with no similarities to the target but its length (22 nucleotides). Oligo B differs in 2 complete nonconsecutive triplets. Oligo C differs in 6 nonconsecutive nucleotides. Oligo 3 differs in 3 nonconsecutive nucleotides. Oligo E represents an oligo with a single nucleotide polymorphism (Table S5). These nonspecific oligos were added to the sample matrix at known concentrations in order to test the linearity of the response of the assay.

**Figure 4:**
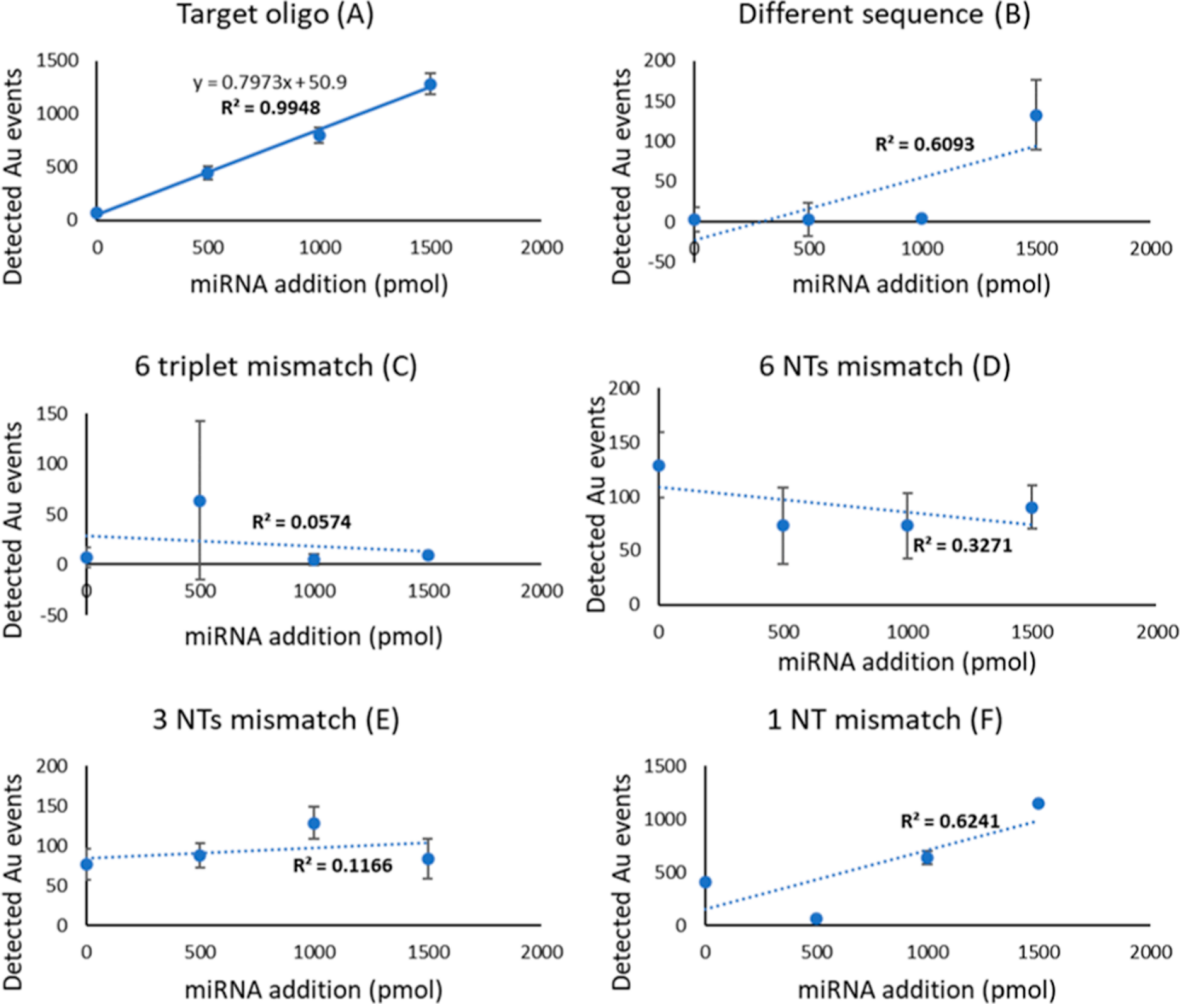
Calibration curves obtained with increasing known concentrations of oligonucleotides that differ in different extents from the target (A), according to Table S5. The lack of linearity with oligos (B–E) and the decreased response with oligo (F) (single nucleotide polymorphism) show the high selectivity of the assay. Error bars show the standard deviation of three replicates for each concentration level.

Figure 4 shows the calibration curves obtained for each of the unspecific oligos. There is a clear linear relationship between the number of gold events detected and the amount of added target (Figure 4A, *R*^2^ = 0.99); there is no linearity when the same assay is performed in the presence of oligos B–F (Figure 4B–F) instead of the target sequence, with values for the correlation coefficient (*R*^2^) of the linear correlation between 0.05 and 0.62, very low response factors, and high irreproducibility. In fact, whereas all concentration levels provide a number of events that are statistically different from any other with the target oligo, many concentration levels are not different from the blanks when using the mismatched sequences. This indicates that the method does not respond to oligonucleotides with a very similar sequence to the target.

### Application to Real Samples: Proof of Concept

The developed strategy was applied to the analysis of three samples of healthy volunteers and used as a proof of concept. Two of them were from sedentary volunteers (less than 700 MET-minutes, according to the GPAQ from WHO). These samples were labeled as Sed_1 and Sed_2. The third sample corresponds to a professional athlete (Ath_1).

A matrix-matched calibration obtained by the addition of known amounts of target oligo to a blood serum pool was performed in order to correlate the number of AuNPs detected with the miRNA concentration in the samples, as previously shown. All samples S1, S2, and A1 were processed in triplicate following the same steps as the calibration standards, obtaining the results shown in Table 2.

**Table 2:** Results Obtained for the Three Samples Used for Quantification^a^

sample code	MET-minutes from GPAQ	miR-16-5p concentration (pmol/mL) (average ±SD)	
		current assay	RT-qPCR
Sed_1	300	2.81 ± 0.06	3.1 ± 0.5
Sed_2	640	1.18 ± 0.28	0.70 ± 0.15
Ath_1	N/A	0.38 ± 0.06	0.30 ± 0.02

a Sed_1 and Sed_2 correspond to sedentary volunteers (a higher score implies higher physical activity). Ath_1 corresponds to a professional athlete. The score for athletes training more than 150 min per day cannot be quantified by the same method. The results obtained with the proposed assay and traditional rt-PCR can be compared in the third and fourth columns.

The results in Table 2 show an inverse correlation between physical activity and the concentration of miR-16-5p in the samples measured with the proposed calibration strategy. Such a correlation is in agreement with previous studies^42,49^ that found a decrease in the expression of miR-16-5p in subjects exposed to increased physical activity, both in humans and other animals.

In order to validate the proposed method, the concentration of miR-16-5p in the same samples was quantified using RT-qPCR. The exponential calibration curve can be seen in Figure S7. As shown in Table 2, despite the totally different quantification procedures, the results obtained by both methods do not show statistical differences in a Student-*t* test (*P* = 0.05), also providing the same trend in the different samples. This shows the potential of the proposed strategy over existing amplification-based strategies.

Besides the matching results, the obtained concentrations for miR16-5p agree with the values previously obtained. However, due to the intrinsic problems of RT-qPCR, most studies do not provide absolute concentrations but only the number of reads for a qualitative comparison. In this regard, previous publications showed a significant overexpression of miR-16-5p in sedentary people compared to this of athletes,^50^ in a similar way as found in this work.

## Conclusions

In this study, we introduce a novel amplification-free miRNA analysis method using magnetic microparticles for capture and preconcentration and gold nanoparticles as labels that amplify the response for ICP–MS detection. Eliminating the need for nucleic acid sequence amplification, our approach streamlines the analysis process while reducing potential sources of error that are common in conventional techniques. Moreover, it circumvents the often challenging considerations of oligonucleotide-nanoparticle conjugation stoichiometry. Importantly, this methodology also demonstrates remarkable resilience to batch-to-batch variations in conjugation processes, which are accounted for in daily calibration.

The high selectivity of the assay, thanks to the complementarity interactions between probes and analytes, has been demonstrated, achieving the possibility of clearly discriminating a 2-base mismatch. Even more, the response to single nucleotide polymorphisms is seriously affected, and these kinds of important sequence variations can also be also discriminated. The developed method, which has been validated against the gold-standard RT-qPCR, can be easily modified to detect any miRNA sequence by changing only the detection and capture probes. This allows for high versatility with minimal optimization for the detection of any miRNA sequence.

## Supporting Information Available

Characterization of the magnetic micro-particles using SEM and energy dispersive X-ray spectroscopy, data showing the optimization of the concentrations of oligonucleotides used for the conjugations, experimental procedure and geometrical considerations, additional data on nano- and micro-particles characterization, data on the optimization steps of the experiment, sequences of oligonucleotides, and comparison of the proposed methodology with other strategies for nucleic acid quantification (PDF)
