# Supplementary material for: Amplification-Free Strategy for miRNA Quantification in Human Serum Using Single Particle ICP–MS and Gold Nanoparticles as Labels
Source: Anal Chem. 2024 Jul 19;96(30):12414–23. doi: 10.1021/acs.analchem.4c01904 (PMC11295127; doi:10.1021/acs.analchem.4c01904)
Supplement: Supplementary file 1 — ac4c01904_si_001.pdf [file ac4c01904_si_001.pdf]

Supporting Information

Amplification-free strategy for miRNA quantification in human serum using single particle-ICP-MS and gold nanoparticles as labels

Sara González Morales[1,3], Carlos López-Portugués[1,3], Manuel Fernández-Sanjurjo[2,3], Eduardo Iglesias-Gutiérrez[2,3], María Montes Bayón[1,3,*], Mario Corte-Rodríguez[1,3,*].

[1]Department of Physical and Analytical Chemistry, Faculty of Chemistry, University of Oviedo, Julián Clavería 8, 33006 Oviedo, Spain.

[2]Department of Functional Biology (Physiology), University of Oviedo, Julián Clavería s/n, 33006 Oviedo, Spain.

[3]Health Research Institute of the Principality of Asturias (ISPA), Av. Hospital Universitario s/n, 33011 Oviedo, Spain.

TABLE OF CONTENTS

Page	Reference	Description
S-2	Text S1	Experimental procedure followed to calculate the number of oligonucleotides bound per nanoparticle.
S-2	Text S2	Geometrical considerations to calculate the maximum number of binding sites on a single gold nanoparticle
S-3	Figure S1	Raw signal intensity for the counting of AuNPs from the analysis of 1.5 nmol of target miRNA. The red line shows the intensity threshold set for the counting.
S-3	Figure S2	Histograms showing the size distribution of over 100 nanoparticles graphically measured in TEM pictures.
S-4	Figure S3	Calibration curves obtained by FIA-ICP-MS for the determination of the number of probes bound to one gold nanoparticle.
S-4	Figure S4	Scanning electron microscopy pictures of the magnetic microparticles.
S-4	Figure S5	Characterization of the magnetic microparticles using energy dispersive X-ray spectroscopy
S-5	Figure S6	Number of gold events detected using different washing strategies
S-5	Figure S7	Calibration curve obtained by RT-qPCR for miR-16-5p quantification in the samples. The R-squared for the fitting was 0.9809.
S-6	Table S1	Sequences of the target, surrogate DNA target and oligo probes used in the assay.
S-6	Table S2	Concentration of oligonucleotide used for the bioconjugation with the gold nanoparticles, compared to the fraction of unbound oligo found after removing the bioconjugated nanoparticles from the suspension.
S-6	Table S3	Concentration of oligonucleotide used for the bioconjugation with the magnetic microparticles, compared to the fraction of unbound oligo found after removing the bioconjugated microparticles from the suspension.
S-7	Table S4	Comparison with SP-ICP-MS based strategies for nucleic acid quantification.
S-8	Table S5	Sequences of the target oligonucleotide and the unspecific oligos A-E used for the selectivity experiment.

Text S1: Experimental procedure followed to calculate the number of oligonucleotides bound per nanoparticle.

The number of oligonucleotide molecules that were bound to the gold nanoparticles was determined in a set of experiments using a flow injection analysis (FIA) setup coupled with ICP-MS detection. 1% HNO_3 was used as carrier at a flow rate of 0.6 $mL \cdot min^{-1}$. The carrier phase was pumped using a peristaltic pump and the sample was injected using an injection valve, model 9125 from Rheodyne (California, USA) fitted with a 20 µL PEEK injection loop (Upchurch Scientific, Washington, USA). All connections to and from the injection system and to the ICP-MS were PEEK tubings.

Gold nanoparticles were quantified by monitoring $^{197}Au^{+}$ in the ICP-MS. For the quantification of the oligonucleotide, P was monitored using the triple quadrupole mode and O_2 as reaction gas to finally detect the $^{31}P^{16}O^{+}$ ion at m/z 47 to avoid the polyatomic interference $^{14}N^{16}O^{1}H^{+}$ on m/z 31. Due to the different concentrations and sensitivity for Au and P, the conjugates were incubated in 0.18 M phosphate buffer for 20 min at room temperature with 1 M dithiothreitol (DTT), a reducing agent that is able to hydrolyse the S-Au bonds that bind the oligo to the surface of the gold nanoparticle, as previously described[40]. Separating the conjugates before the analysis allowed to apply much lower dilution factors to the DNA fraction to quantify the P than those applied to the NP fraction for Au quantification.

The system was calibrated using Au and P elemental standards. The standards were injected, in triplicates, in the same experimental conditions as the samples. Peak areas were correlated with the known concentrations using a linear regression to build the calibration curve. The triplicate peak areas of three independent incubations of the oligonucleotide with the nanoparticles were obtained and interpolated in the calibration curve to obtain Au and P concentrations.

Au and P concentrations were translated, after adequate calculations and taking into account all dilution factors, into number of gold nanoparticles (considering 22 nm AuNPs) and number of oligonucleotide molecules (considering one molecule of oligonucleotide has 35 atoms of P in the structure), respectively. After this, the ratio of oligonucleotide molecules:AuNPs provided a good estimation of the stoichiometry of this conjugate.

In order to consider and correct possible matrix effects, the FIA-ICP-MS quantification was repeated applying standard additions of phosphate standards for the oligonucleotide (Figure S3). The two slopes were not statistically different, therefore discarding the influence of matrix effects in this determination.

Text S2: Geometrical considerations to calculate the maximum number of binding sites on a single gold nanoparticle

This is a plausible stoichiometry if some geometrical considerations are accounted. Firstly, the surface area of a 22 nm diameter gold nanoparticle can be easily calculated as 1520.5 nm^2. The whole nanoparticle will be made of pure gold, which crystallizes in a face-centred cubic structure. The surface of the nanoparticle can be approximated as totally formed by faces of gold unit cells. Since the area of these cell faces can be calculated as 0.22 nm^2 from the atomic radius of gold, the surface occupation of one unit cell's face can be calculated as the surface of 2 atoms of gold divided by the total surface, which results in a surface occupation of 78.5%. Considering this occupation, the surface of the nanoparticle would contain a total of 13720 atoms of gold, each of them available to form one covalent bond with a thiol group of the reporter oligonucleotide. However, the number of oligonucleotides that will finally bind to the nanoparticle must be lower due to (i) steric and charge effects of the oligonucleotides; and (ii) not all oligonucleotide molecules will adopt a position that is perpendicular to the surface of the nanoparticle, impairing the binding of other molecules in a bigger area than the given by the mere steric and charge effects.

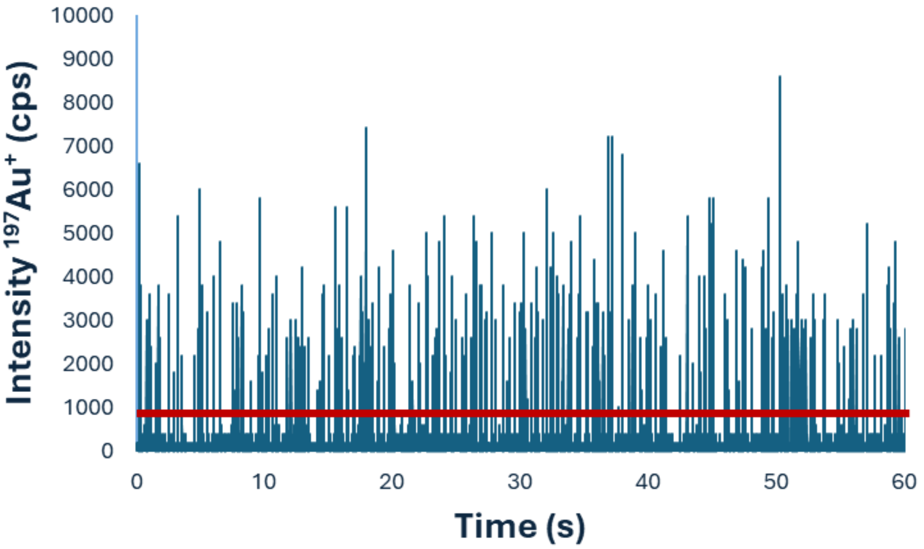

Figure S1: Raw signal intensity for the counting of AuNPs from the analysis of 1.5 nmol of target miRNA. The red line shows the intensity threshold set for the counting.

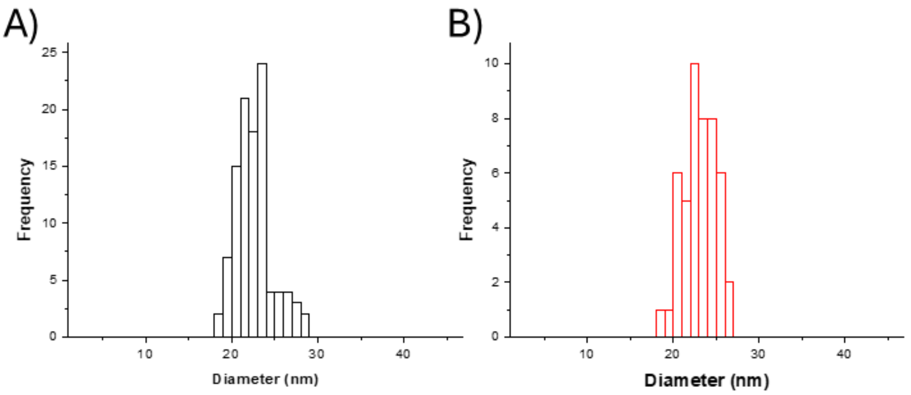

Figure S2: Histograms showing the size distribution of over 100 nanoparticles graphically measured in TEM pictures.

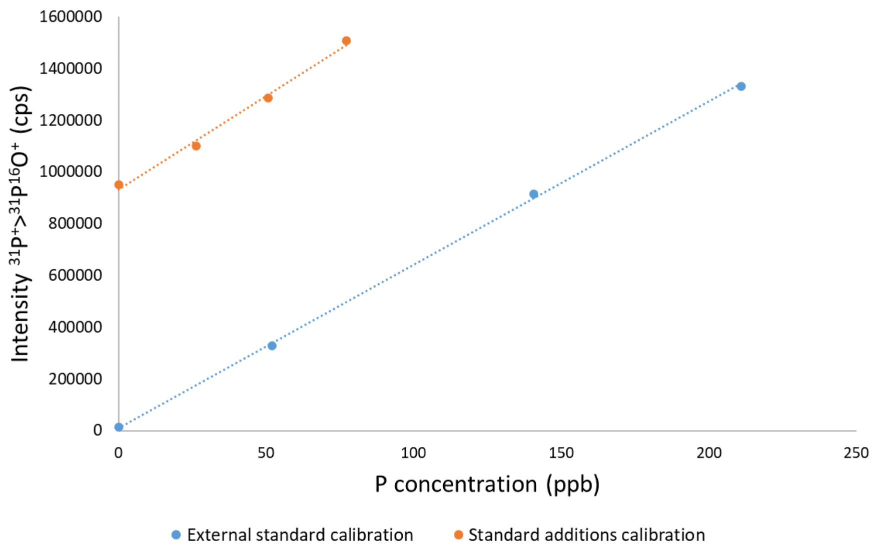

Figure S3: Calibration curves obtained by FIA-ICP-MS for the determination of the number of probes bound to one gold nanoparticle. Standard additions (orange) and external standards (blue) provided the same slope of the linear regression, discarding the existence of matrix effects.

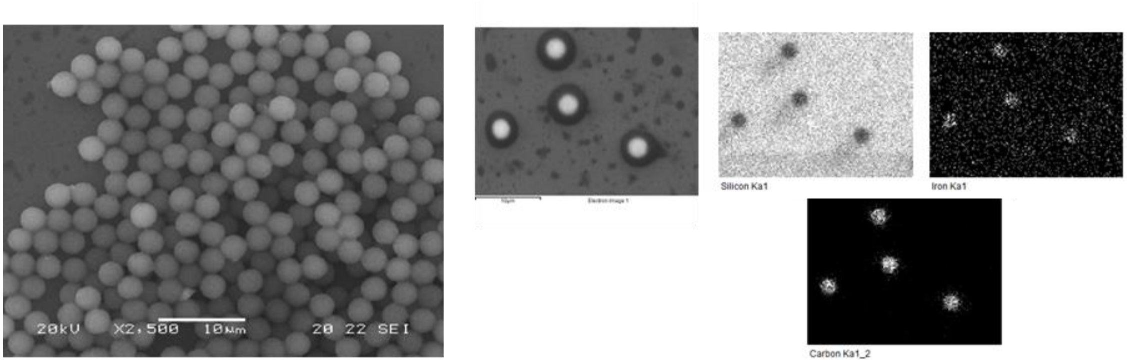

Figure S4: Scanning electron microscopy pictures of the magnetic microparticles. Scale bar is 10 μm. At the right hand side, images corresponding to the emission line of silicon, iron and carbon.

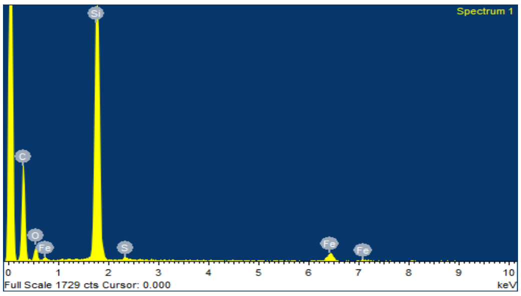

Figure S5: Characterization of the magnetic microparticles using energy dispersive X-ray spectroscopy

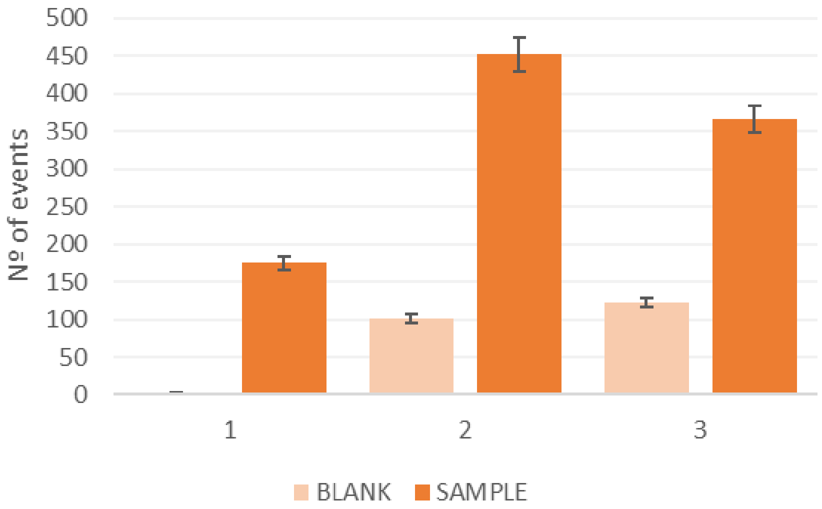

Figure S6: Number of gold events detected using different washing strategies. Number 1 corresponds to moving the sample to a new Eppendorf after the second wash. Number 2 corresponds to increasing the number of washing steps to 4. Number 3 corresponds to decreasing the concentration of reporter probe.

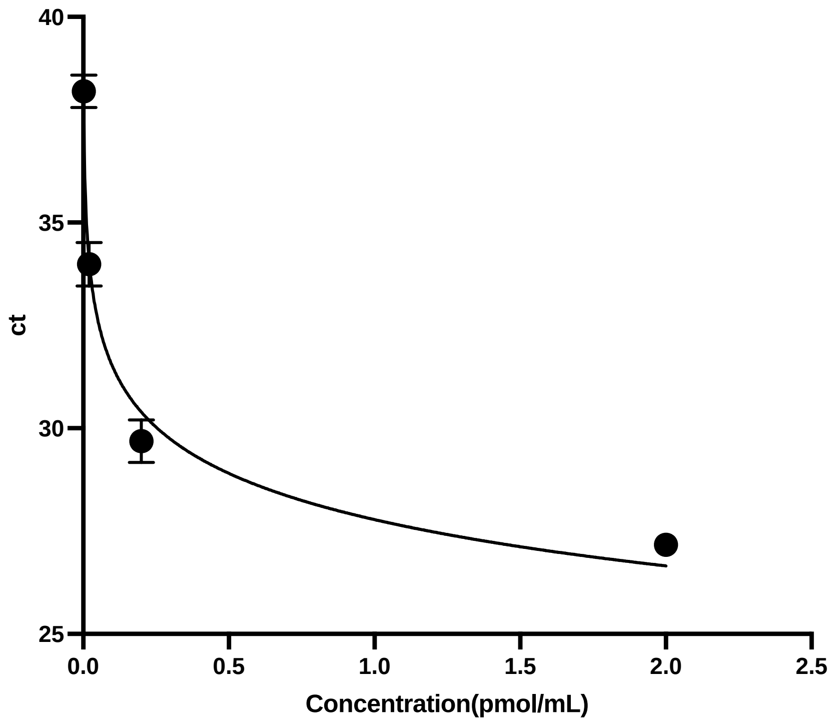

Figure S7: Calibration curve obtained by RT-qPCR for miR-16-5p quantification in the samples. The R-squared for the fitting was 0.9809.

Table S1: Sequences of the target, surrogate DNA target and oligo probes used in the assay.

Sequence Nr.	Name	Sequence
(1)	miR-16-5p (target)	5' - UAG CAG CAC GUA AAU AUU GGC G – 3'
(1*)	miR-16-5p (surrogate DNA target)	5' – TAG CAG CAC GTA AAT ATT GGC G – 3'
(2)	Capture oligo (biotinylated)	5' – T TTA TAA CCG CAA $(AAA)_7$ – BIOT – 3'
(3)	Reporter oligo (thiolated)	5' – THIOL - $(AAA)_8$ ATC GTC GTG CA – 3'

Table S2. Concentration of oligonucleotide used for the bioconjugation with the gold nanoparticles, compared to the fraction of unbound oligo found after removing the bioconjugated nanoparticles from the suspension.

Oligo concentration for incubation (ng/µL)	Fraction of unbound oligo (%)
3.22	<LOD
6.62	15.1
32.66	30.6
64.24	38.9
124.34	45.9
254.41	62.0

Table S3. Concentration of oligonucleotide used for the bioconjugation with the magnetic microparticles, compared to the fraction of unbound oligo found after removing the bioconjugated microparticles from the suspension.

Oligo concentration for incubation (ng/µL)	Fraction of unbound oligo (%)
259	< LOD
517	0.95
1035	0.87
1552	0.94
2070	0.87

Table S4: Comparison with SP-ICP-MS based strategies for nucleic acid quantification.

Target nucleic acid	Target length	Type of sample	Counting/aggregation strategy	Extraction/amplification steps	Natural/spiked	Limt of detection	Reference
miRNA	22 nt	Human serum	Counting	No	Natural	10.7 pM	Proposed
DNA	30 nt	Water/not specified	Aggregation	No	Spiked	1 pM	29
DNA (SARS-CoV-2 and H3N2)	Complete gene (30 nt target region)	Saliva and urine	Aggregation	No	Spiked	1.5 pM	30
DNA (malaria) and miRNA	50 nt and 23 nt	Human serum	Aggregation	No	Spiked	10 pM	31
				Amplification	Spiked	0.1 pM	31
DNA (HIV, HAV, HBV)	28-33 nt	Artificial	Counting	No	Spiked	1 pM	32
DNA (E. coli)	48 nt	Milk	Counting	No	Spiked	10 fM	34
miRNA	22-24 nt	Blood serum	Counting (negative)	Extraction	Natural	50 amol	35

Table S5. Sequences of the target oligonucleotide and the unspecific oligos A-E used for the selectivity experiment. The degree of similarity is expressed in a scale of increasing difference from 1 (the target oligo) to 6 (a totally different sequence). The different nucleotides with respect to the target oligo have been underlined.

Name	Sequence	Degree of similarity
Target oligo - A	5’ – TAG CAG CAC GTA AAT ATT GGC G – 3’	1
B	5’ – CTA GCT ACC GAT ATC TGT CTA T – 3’	6 (totally different)
C	5’ – TAG GCC CAG GTA CTG ATT GGC G – 3’	5 (2 different triplets)
D	5’ – TAG TAG CGC ATA TAT ACT CGC G – 3’	4(6 different nucleotides)
E	5’ – TAG CCG CAC GCA AAT AAT GGC G – 3’	3 (3 different nucleotides)
F	5’ – TAG CCG CAC GTA AAT ATT GGC G – 3’	2 (1 different nucleotide)
